# Flexible Tactile Sensor Array Based on Aligned MWNTs-PU Composited Sub-Microfibers

**DOI:** 10.3390/mi9050201

**Published:** 2018-04-24

**Authors:** Weiting Liu, Xiaoying Cheng, Xiaodong Ruan, Xin Fu

**Affiliations:** 1 State Key Laboratory of Fluid Power & Mechatronic Systems, Zhejiang University, No. 38 Zheda Road, Hangzhou 310027, China; liuwt@zju.edu.cn (W.L.); xdruan@zju.edu.cn (X.R.); xfu@zju.edu.cn (X.F.); 2 Faculty of Mechanical Engineering and Automation, Zhejiang Sci-Tech University, No. 928 Second Avenue, Hangzhou 310018, China

**Keywords:** flexible tactile sensors, sub-microfiber array, nanocomposite, carbon nanotubes, electrospinning

## Abstract

This present paper describes a novel method to fabricate tactile sensor arrays by producing aligned multi-walled carbon nanotubes (MWNTs)-polyurethane (PU) composite sub-microfiber (SMF) arrays with the electrospinning technique. The proposed sensor was designed to be used as the artificial skin for a tactile sensation system. Although thin fibers in micro- and nanoscale have many good mechanical characteristics and could enhance the alignment of MWNTs inside, the high impedance as a consequence of a small section handicaps its application. In this paper, unidirectional composite SMFs were fabricated orthogonally to the parallel electrodes through a low-cost method to serve as sensitive elements (SEs), and the impedances of SEs were measured to investigate the changes with deformation caused by applied force. The particular piezoresistive mechanism of MWNTs disturbed in SMF was analyzed. The static and dynamic test results of the fabricated tactile sensor were also presented to validate the performance of the proposed design.

## 1. Introduction

To simulate the human skin, which has four types of mechanoreceptors to archive high spatial definition and keep flexibility and compliance simultaneously, extensive research has been conducted on developing different kinds of transduction methods for tactile sensors that can be embedded in the structure of a robot or prosthetic hand [1,2,3,4]. Among these mechanisms, the piezoresistive method is prevalent for its simple and low-cost preparation process with good flexibility [5]. Moreover, piezoresistive composite materials made up of the polymeric matrix and conductive fillers have been widely studied over the last decades because of their higher sensitivity and higher compatibility with complex curves than rigid materials such as microelectromechanical systems (MEMS) piezoresistors [6,7,8]. Although the composites are intrinsically sensitive and flexible, the bulky size (especially in the thick direction) of these materials would impede the full utilization of these good properties [9]. In contrast, the form of fine fiber can improve the sensitivity and flexibility of materials [10,11], and unlike direct drawing, template synthesis, self-assembly and phase separation techniques, electrospinning is a more effective and convenient method able to fabricate polymer-based composite fibers in various diameters [12]. However, the nano- and microfibers of functional composites are well applied in many areas, such as biological and chemical sensors [13], whereas the reports about the tactile sensing are few [14,15].

Therefore, to develop a highly sensitive, simply structured and low-cost tactile sensing device, this paper presents a novel proposal of a flexible tactile sensor based on sensitive composited SMF arrays fabricated by a modified electrospinning method. The composite here is a dielectric polymer with conductive particles filled. The electrical resistivity of this material would drop continuously when the volume fraction of dopant increases until the percolation threshold is reached. At this point, the resistance of the composite changes dramatically along with the variation of external force applied on it [16]. Although the associated theory in bulk size has already been extensively studied, the counterpart in slim structure has not been reported other than morphological analysis [17,18]. In this work, conductive polymer composite is prepared in aligned thin fibers of sub-micrometer scale to archive highly orientated arrangement of conductive fillers. Unidirectional composite sub-microfibers (SMFs) are orthogonal to the parallel electrodes to form resistance networks to decrease the high impedance of one single composite fiber. Then two sets of n SEs parallel arrays are perpendicular to each other to form a matrix so that 2*n* sensitive elements (SEs) can make both electrical connection and electronic interface much easier than *N* × *n* SEs. Meanwhile, SMFs are developed by electrospinning process modified by rotating technique which has a much simpler processing than other solutions of producing the slim structure.

In the previous study, it was found that a polymer integrated with carbon nanotubes could achieve good piezoresistive performance because of the property of the latter material [19]. In this paper, multi-walled carbon nanotubes (MWNTs) are distributed into polyurethane (PU) to realize the tactile sensor. By a modified electrospinning technique, composite fibers are fabricated onto a flexible printed circuit board (FPCB), which is demonstrated later. The resistivity-force characteristics of these sensors are investigated. Other relevant data such as static and dynamic properties are also shown. The mechanism of the sensing process and the structure of the proposed sensor is described in Section 2. Section 3 is about the fabrication of sensing elements through the modified electrospinning method. Then, the test results are shown and discussed in Section 4.

## 2. Sensor Mechanism and Design

### 2.1. Mechanism Analysis

In this work, sensitive elements are made of PU with doped MWNTs. Carbon particles are utilized as one of the most common conductive filling materials for their high electrical conductivity and stability. When approaching percolation threshold, MWNTs can not only improve the sensitive of piezoresistive composite much more than other allotropes but also decrease the influence on the impedance from ambient temperature [20]. A large amount of research has already been reported about the application of MWNTs/polymer composite in developing strain and force sensors in which the sensitive elements are fabricated in bulk size [6,21,22]; however, in this paper, a novel tactile sensor with the sensitive element in thin aligned fibers form is described. Thin fiber in sub-micrometer diameter not only constrains the dispersion of MWNTs and increases their alignment as well as electrospinning progress does [23], but also the drum collector arranges the polymer fibers parallel so that MWNTs in different fibers remain in the same orientation which makes SEs show anisotropy in electricity (see Section 4).

**Figure 1:**
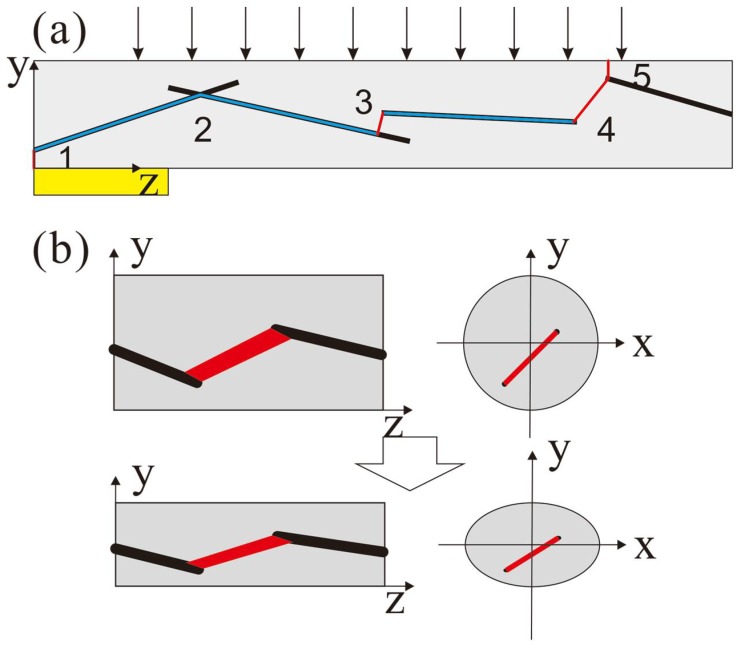
(**a**) A typical dispersion of multi-walled carbon nanotubes (MWNTs) (simplified as long rods) in a polyurethane (PU) fiber, where the blue line indicates the electrical path along the surface of MWNT and red one between two MWNTs; (**b**) The change of electrical path between two MWNTs when the fiber is deformed by applied force.

The electrical conductivity of sensitive fiber is mainly decided by the distance between the conduct fillers (the conductivity of MWNTs is 10^4^ S/m, whereas that of PU is usually less than 10^−12^ S/m). However, different from the stretching situation, SMFs in the proposed sensor are compressed in the radial direction as shown in Figure 1a. This drawing also demonstrates a typical dispersion of MWNTs (simplified as long rods) in a PU fiber with an electric passage lined out. The blue lines are the passages along the surface of MWNTs, and red ones are the passages in the polymer that dominate the resistance of the composite fiber. The later passages could be divided into two kinds: the one connects to the outside of the fiber (as 1 and 5 in Figure 1a) and the other connects to the inside of the fiber (as 2, 3 and 4 in Figure 1a). Situation 2 shows that two MWNTs are contacted or within the tunneling distance, while situation 3 shows that two MWNTs are overlapped and four are the separated in the longitudinal direction. Considering the longitudinal distance between MWNT and electrode or between MWNTs in different fibers, situations 1 and 5 could be classified into situation 3 or 4. Thus, the resistance of one fiber could be described as

(1) $$R_{\mathrm{fiber}}=\sum_{i=1}^{j}R_{\mathrm{cnt}i}+\sum_{i=1}^{k}R_{ci}+\sum_{i=1}^{m}R_{oi}+\sum_{i=1}^{n}R_{si}$$

where *R*_cnt*i*_ is the resistance of one MWNT along its surface, *R*_c*i*_ is the resistance in situation 2, *R*_o*i*_ is situation 3, *R*_s*i*_ is situation 4; *j*, *k*, *m*, and *n* are the amounts of these resistances respectively. Because the former two parts are smaller than the latter two by orders, the whole resistance could be estimated by formula

(2) $$R_{\mathrm{fiber}}\approx \sum_{i=1}^{m}R_{oi}+\sum_{i=1}^{n}R_{si}$$

As in Figure 1b, once the fiber is affected by a uniform load, the radius in the *y* direction will compress and that in the *x* direction will elongate. However, because of the high aspect ratio of SMF, the length in the *z* direction will barely change, and it can thus be ignored, simplifying the analysis. This deformation causes two opposite effects on the whole resistance of the composite fiber: on one hand, according to the law of resistance, the decrement of the section’s area (the elongation in the *x* direction is shorter than the compression in the *y* direction for Poisson’s ratio) such as in situation 3 will increase the resistance; on the other hand, the two parts in situation 2 will decrease because the distance between two MWNTs in the *xy* plane becomes smaller whereas the distance in the *z* direction changes little, which possibly causes dramatic local resistance change owing to the distance variation in the range of quantum-tunnelling effect in case of oriented arrangement of MWNTs. The predominated function of the two contradictories determines the overall resistance change. When the ratio of MWNTs is near percolation, the resistance of composite fiber will drop significantly under load.

### 2.2. Structure Design

**Figure 2:**
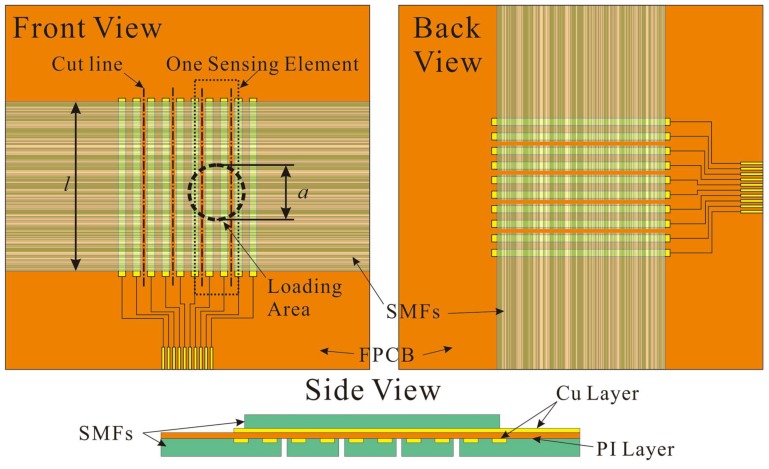
The schematic demonstration of the proposed flexible tactile sensor with two orthogonal unidirectional composite sub-microfiber (SMFs) layers on both side of flexible printed circuit board (FPCB). One SE consisting of parallel SMFs between two electrodes is indented with a round contact area as in the figure.

There are two layers of aligned composite SMFs in the proposed flexible tactile sensors. These layers are mutually perpendicular and fabricated on different sides of an FPCB (Figure 2). A serial of parallelized Cu strips on the polyimide (PI) film serve as electrodes which segment the sensing layer. PI and Cu layers are 20 μm and 15 μm thick respectively. The Cu strip is 0.25 mm width and the central distance of electrodes (CDoE) is set at 0.5 mm, 1.0 mm, and 2.0 mm to prepare sensors with different spatial resolutions. The detail of the fiber structure will be discussed in Section 3.1. It is very important that, between Cu strips, parallel resistor nets are formed to lower the resistance and increase the measurability of composite fiber. As in Figure 2, several cuts are made on the SMFs layer every two electrodes to separate each SE so as to decrease the coupling effect between adjacent ones. Considering an SE affected by a uniform stress, the impedance measured is

(3) $$R_{m}^{\prime }=\frac{a\left(l-a\right)R_{\mathrm{fiber}}R_{\mathrm{fiber}}^{\prime }}{\left(l-a\right)R_{\mathrm{fiber}}+aR_{\mathrm{fiber}}^{\prime }}$$

where *R^′^*_fiber_ is the resistance of the fiber in the loading area, *R*_fiber_ is the resistance of the fiber out of the loading area, *l* and *a* are the lengths of the SE and diameter of loading area respectively which can also indicate the quantity of fibers. Each SE array of one side can detect the force distribution in one dimension, and the information about force location and amplitude can be derived through combining the responses of SEs from both sides.

### 2.3. Signal Acquisition

**Figure 3:**
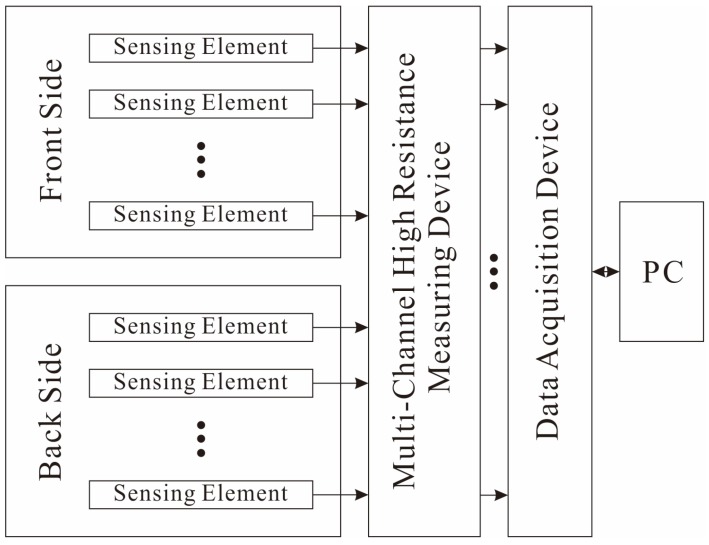
Diagram of the data acquisition system for the proposed sensor.

Figure 3 is the diagram of the data acquisition system. However, the normal multiplex circuits cannot obtain the current signal from the SE because of the high resistance. Thus, the resistance signals of piezoresistive arrays are converted to voltage signals by a specially developed 16-channel high resistance measuring device. Then, the signals are sent to the acquisition board (NI-6394, National Instruments Corporation, Austin, TX, USA) and the data are analyzed and displayed on a computer. By changing the mass ratio of fillers in composite, the average impedance between two electrodes could be adjusted to the scale of 10^9^ Ω (see Section 3.1). Thus, the high resistance measuring device is designed to detect resistance variation ranging from 1.0 × 10^8^ Ω to 0.4 × 10^10^ Ω.

## 3. Development of Sensor

### 3.1. Preparation of Composited Material

The MWNTs used in this paper are Dimethylformamide (DMF) based on dispersion with 7.8 wt % of solid (Timesdisper, TNDDM, Chengdu Organic Chemicals, Chengdu, China), and the PU is Elastollan 1185 A from BASF SE (BASF Polyurethanes GmbH, Lemförde, Germany). The densities of the MWNTs and PU are 1.8 g/cm^3^ and 1.12 g/cm^3^, respectively. The outside diameter of the MWNTs is 50 nm and the length is around 10~20 μm. To increase the spinnability of the composite, we have chosen tetrahydrofuran (THF, Sinopharm Chemical Reagent, Shanghai, China) as the solvent so that PU is kept at 15 wt % of the solution. To maximize the sensitivity of composite, the volume ratio of filler and polymer should be close to percolation threshold. This value is converted to mass ratio for convenience.

Differently to other conductive fillers, MWNTs can be treated as long rods with a large aspect ratio. According to Celzard et al.’s work [24], the excluded volume method has good efficiency in estimating the critical concentration of material in this shape. The critical concentration, which is the volume fraction of the filler in composite, can be calculated by

(4) $$\phi_{c}=1-\exp\left(-\frac{\langle V_{\mathrm{ex}}\rangle V_{\mathrm{cyl}}}{\langle V_{e}\rangle }\right)$$

where *V*_cyl_ is the volume of filler and can be derived from the following equation with assuming that MWNTs are long rods (*l*) with hemispherical ends (*r*):

(5) $$V_{\mathrm{cyl}}=\frac{4}{3}\pi r^{3}+\pi lr^{2}$$

<*V*_e_> represents the average exclude volume of this rod-like filler and it is given by

(6) $$\langle V_{e}\rangle =\frac{32}{3}\pi r^{3}+8\pi r^{2}l+\pi rl^{2}{\langle \sin\gamma \rangle }_{\mu }$$

**Figure 4:**
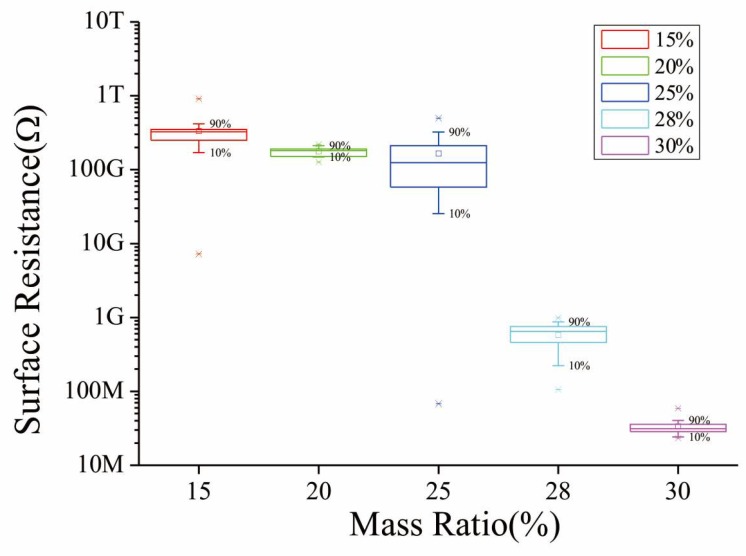
The surface resistance of one sensitive element (SE) with the mass ratio varying from 15% to 30%.

<sin*γ*>_*μ*_ is an indication of the orientation degree of the fillers and complex to be calculated; however, there are two extreme cases of this volume: γ=π/4 for randomly aligned fillers and γ=0 for parallel aligned ones. *<V*_ex_*>* is the total excluded volume and same as <sin*γ*>_*μ*_: 1.4 for randomly and 2.8 for parallel [25]. Thus, the critical concentration can be as high as 29.53% when the MWNTs are totally paralleled. To search for this volume ratio of the composite SMF, we have prepared and exploited a series of different solutions for the fabricating process described in Section 3.2 (CDoE is 0.5 mm and the width of fiber film is 25 mm). The surface resistance is measured by a high resistance meter (ZC-90G, Shanghai Taiou Electronic, Shanghai, China) and shown in Figure 4, and the abscissa is the mass ratio for convenience. It is obvious that the critical concentration is located between 25% and 30% mass ratio, i.e., 15.5% and 18.6% volume ratio. Considering the resistance of SE decreases when force is applied, the mass ratio of the composite is chosen at 25% in order to reach reliable resistance measurement.

### 3.2. Fabrication of Sensitive SMF by Modified Electrospinning

The electrospinning method is an efficient technique to fabricate fine polymer fibers with a diameter ranging from several nanometers to micrometers [26]. However, the randomness of fibers’ arrangement is a significant defect of this method. To overcome this problem, many researchers have reported several modified techniques [27,28,29,30]. In this paper, a drum collector [30] is implemented to produce thick and large fine fiber film so that its resistance could be decreased effectively [31].

**Figure 5:**
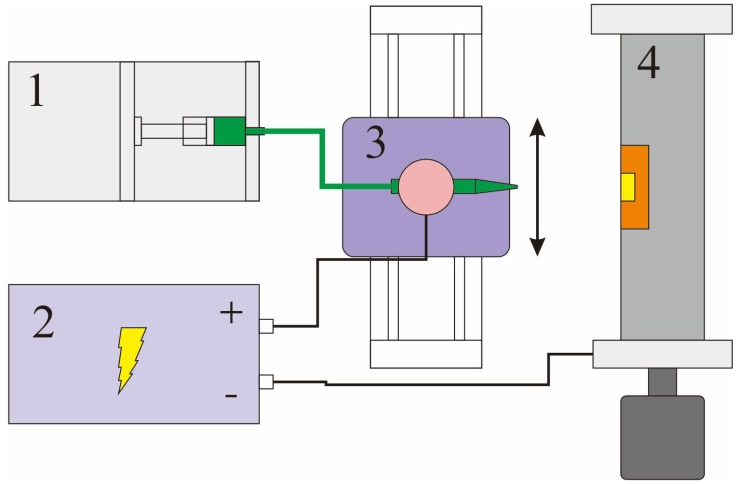
The diagram of fabrication system of aligned SMFs on FPCB based on electrospinning device. Number 1 is a syringe pump, 2 is a high voltage generator, 3 is a linear motion stage and 4 is a motor-driven aluminum drum with FPCB attached.

The diagram of the fabrication system is shown in Figure 5. The pipette with a 1 mm diameter outlet is mounted on a motion stage which is jogging at 1 mm/s with a stroke of 25 mm during the fabricating process. An aluminum drum with a diameter of 75 mm is driven by a step motor to 5000 rpm and the FPCB is attached to its surface with its electrodes parallel to the axis. It is important to connect all the electrodes to the drum to increase the effect of alignment of the fibers [29]. A syringe pump is connected to the pipette and supplying the composite solution at 0.5 mL/h. The distance between the tip and drum is 6 cm and 12 kV voltage is applied.

**Figure 6:**
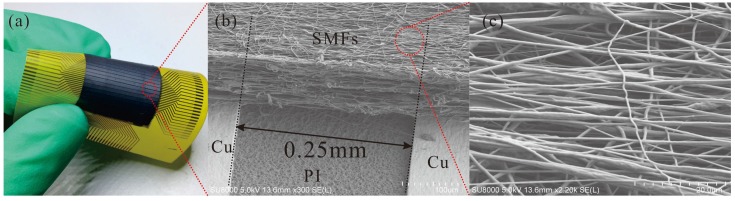
(**a**) A photo of proposed flexible tactile sensor with 0.5 mm central distance of electrodes (CDoE); (**b**) An SEM image of SMFs between two Cu electrodes; (**c**) A higher amplification SEM image of SMFs to show the alignment.

**Figure 7:**
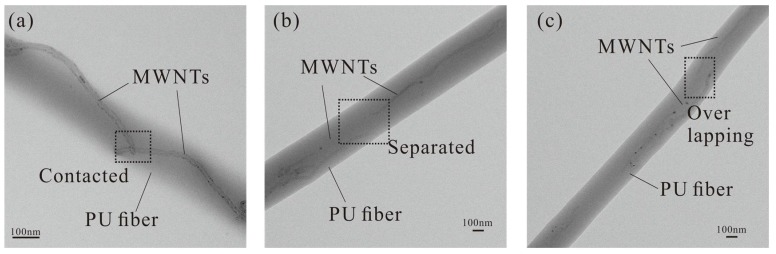
Transmission electron microscope (TEM) images to show positional relations between MWNTs in PU SMF: (**a**) Contacted MWNTs; (**b**) Separated MWNTs; (**c**) Overlapping MWNTs.

It is difficult to determine the thickness of the fiber film during the fabrication. Therefore, electrospinning time is chosen as the factor to control the thickness. After the fabrication on one side is finished, the FPCB is turned over and rotated 90° before reattached to the drum, and then the other side begins to collect. The photo of a fabricated flexible tactile sensor is shown in Figure 6a. A scanning electron microscope (SEM; SU8010, Hitachi, Tokyo, Japan) image of SMFs between two electrodes in Figure 6b is clearly shown the detail designed structure, and the collected fibers are distributed in an acceptable alignment. However, the structure is not totally regulated and the main reason is the high conductivity of electrospinning solution which increases the bending instability of jet [32]. Investigation of the high magnification SEM photo (Figure 6c) revealed to us that the diameters of composite fibers were mainly under 1 μm in the sub-micrometer scale. Also, three transmission electron microscope (TEM; CM200, Philips, Amsterdam, The Netherlands) images of a single composite SMF in Figure 7 shows the typical situation 2, 3 and 4 of MWNTs dispersion described in Section 2.1 which dominate the resistance change while a force is applied. To protect the sensitive fibers, we have applied a polyimide (PI) layer (7413D, 3M Company, Maplewood, MN, USA) with the thickness of 60 μm over the SMF layer.

## 4. Test and Discussion of Results

### 4.1. Piezoresistivity of Single SE

**Figure 8:**
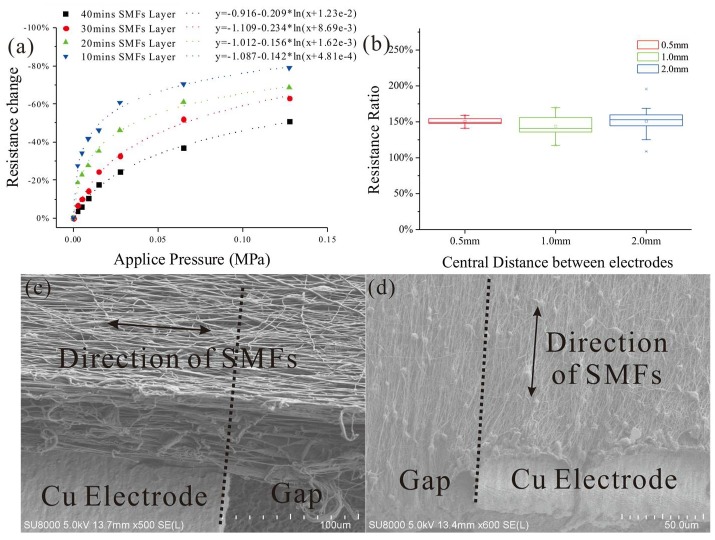
(**a**) The resistance change of SE with different thickness of SMFs layer under applied weight (measured in 10~15 s after weight placed); (**b**) The resistance change ratio comparison between SMFs perpendicular and parallel to electrodes with different CDoE; (**c**) SEM image of SMFs perpendicular to electrodes; (**d**) SEM image of SMFs parallel to electrodes.

To measure the piezoresistivity of the parallel SMF structure between two electrodes, a polymethylmethacrylate (PMMA) disk with a diameter of 10 mm is placed on the composite material of the proposed sensor and the SE under the center of the disk is observed. The resistance changes of SEs with different fabrication time are measured by the high resistance meter when the readout is stable after different weights are placed on this square. The test results drawn in Figure 8a show that the impedance of SMF decreases dramatically when the weight starts to increase (the initial weight is 20 grams) and, after a period of fast dropping, it slowly reaches a flat area where adding weight (increased to over 1000 gram) influences it little because the Young’s modulus of PU increases greatly along with the increasing of deformation. To fit the results from SMFs fabricated in 10 min, a logarithm function is used for the resistance change:

(7) $$y=-1.087-0.142\ln\left(x+4.81\times 10^{-4}\right)$$

According to Fechner’s law [33], for human perception, the relation between stimulus and perception is a logarithm which just fits the sensitive curve of proposed tactile sensor. This property grants the sensor high sensitivity to distinguish the small differences in low force area and a large range of measurement.

By changing the electrospinning time, a series of SMF layers of different thicknesses is produced and the increase of the thickness (represented by electrospinning time) reduces the change rate of resistance obviously, as in Figure 8a. The main reason for this reduction could be that the thicker the SMF layer is, the less the decrement ratio of thickness under the same weight would become because the strain of the elastic layer that counterbalances the pressure stays the same.

To study the effect of parallel resistors networks formed by unidirectional SMFs orthogonal to the parallel electrodes, tactile sensors with the direction of SMFs along the parallel electrodes are prepared and served as comparatives. The comparisons between resistances of SEs from different sensors are drawn in Figure 8b while the SEM images of SMFs in two different arrangements are shown in Figure 8c,d. The impedances from counterparts are higher than from the proposed sensor and this disparity is maintained at around 150% as the CDoE enlarges, which means the proposed structure has a persistent effect on decreasing the resistance of SE. This phenomenon could be explained as that the MWNTs in the SMFs orthogonal to the electrodes could form the electrical paths as described in Section 2.1 whereas electrical paths in the SMFs parallel to the electrodes would mainly depend on situations 1 and 5 which have higher impedances than the rest.

### 4.2. Static and Dynamic Validation Test

**Figure 9:**
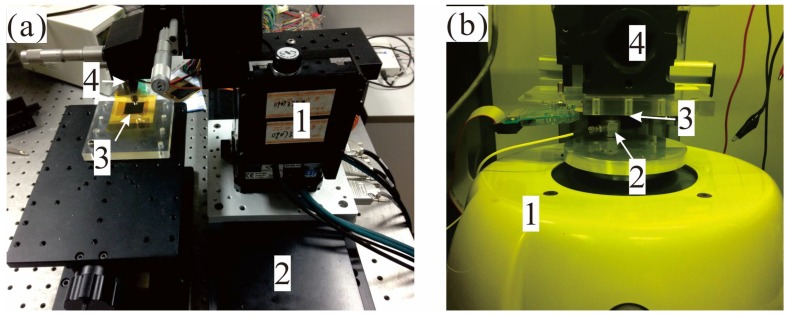
(**a**) A photo of static test equipment, where 1 is an auto positioner, 2 is a linear motion stage, 3 is proposed sensor and 4 is a static force sensor; (**b**) A photo of dynamic test equipment, where 1 is a vibration exciter, 2 is a dynamic force sensor, 3 is proposed sensor and 4 is manual positioner.

Experiment tests to validate the prototype flexible tactile sensor can be divided into two kinds by the equipment setups: static validation test and dynamic validation test. The former implements a static indentation test and sliding test with the combination of an auto positioner (VP-25XYZL, Newport, Irvine, CA, USA) and a linear motion stage (LM-600, Newport) while the latter performs a dynamic indentation test with a vibration exciter (V406, Brüel & Kjær, Nærum, Denmark, controlled by Spider-81B controller from Crystal Instruments, Santa Clara, CA, USA) as the photos shown in Figure 9.

**Figure 10:**
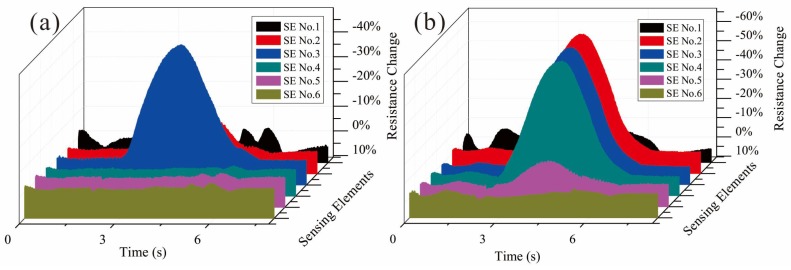
The results (filtered by 20 Hz low-pass) of indentation test with different contact disks on parallel SEs (CDoE is 1.0 mm): (**a**) 5 mm diameter; (**b**) 10 mm diameter.

**Figure 11:**
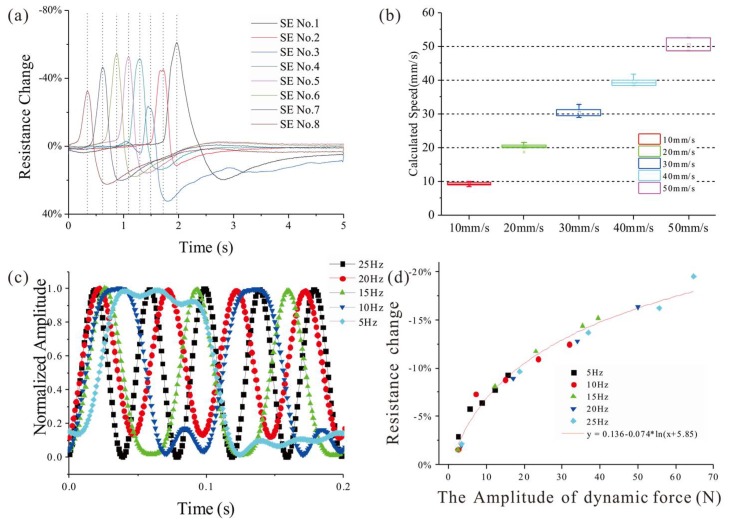
(**a**) Output of parallel SEs (CDoE is 1 mm) under a sliding of 10 mm/s filtered by 30 Hz low-pass; (**b**) Calculated speed from the time difference of output’s peak between adjacent SEs; (**c**) Normalized output of a SE under sine indentations with different frequencies filtered by 30 Hz low-pass; (**d**) The responses of the SE to sine indentations with different frequencies.

In the static test, parallel SEs on one side of the FPCB are considered. During the static indentation test, a series of PMMA round disks with different diameters is set between indenter and sensor. The resistances of the SMFs under the disk will decrease at the present of indentation force and the change of impedance between two electrodes should follow with Equation (3). The results in Figure 10 demonstrate that the structure of parallel SEs has a good spatial discrimination in one direction. For the sliding test, a polydimethylsiloxane (PDMS) cylinder is attached to the end of the indenter to serve as an elastic buffer and driven by the linear motion stage with different velocities. A typical output of the parallel SEs under a sliding of 10 mm/s is drawn in Figure 11a, and by measuring the time differences between the adjacent SEs and considering the distance between them, the sliding speed could be calculated from the signals. The calculation results of varying speed from 10 mm/s to 50 mm/s are drawn in Figure 11b and compared with the real speed to show the accuracy of the estimation by proposed sensor.

In the dynamic test, the indentation force is set as a sine wave with varying frequencies from 5 Hz to 25 Hz while the amplitude is invariant to validate the frequency-domain performance of the tactile sensor. Figure 11c draws the filtered signals of one SE in a 0.2 s period under the indentation force of 5 different frequencies, and for easy to compare, all the amplitudes are normalized. Figure 11d shows the responses of the SE to the indentation force of different frequencies. From the curves, we could find that the proposed sensor has a good response to the dynamic force with the frequency under 25 Hz.

## 5. Conclusions

Composite SMF has both the piezoresistive property of conductive composite and good mechanical characteristics from its sub-micro scale. The proposed structure of unidirectional SMFs orthogonal to the parallel electrodes could decrease the impedances of SEs while increasing the piezoresistive property of them. The fabrication of the tactile sensor proposed in this paper is simple to be realized, and the flexibility of this tactile sensor is obvious, as shown in the photo. The prototype sensors are fabricated and their static and dynamic performances are also tested. The proposed sensor could detect applied static force changing within 10 N and has a minimum sensitive to 0.2 N; it also could measure the speed of sliding in the direction perpendicular to the electrodes. For dynamic testing, this sensor could follow the sine indentation with frequencies from 5 Hz to 25 Hz. However, the surface resistance of SE is still too large (~10^9^ Ω) which produces very small signals that hinder the development of signal acquisition circuits and weaken the capacity of resisting interference. Further research on this flexible tactile sensor should focus on increasing the conductivity of SMF, and also another future work would be to mount the sensor onto a prosthetic and grant closed loop control on the task of grasping subjects.

## References

[1] Kappassov Z., Corrales J.-A., Perdereau V. (2015). Tactile sensing in dexterous robot hands—Review. Robot. Auton. Syst..

[2] Wang X., Dong L., Zhang H., Yu R., Pan C., Wang Z.L. (2015). Recent progress in electronic skin. Adv. Sci..

[3] Chortos A., Liu J., Bao Z. (2016). Pursuing prosthetic electronic skin. Nat. Mater..

[4] Zou L., Ge C., Wang Z., Cretu E., Li X. (2017). Novel tactile sensor technology and smart tactile sensing systems: A review. Sensors.

[5] Stassi S., Cauda V., Canavese G., Pirri C.F. (2014). Flexible tactile sensing based on piezoresistive composites: A review. Sensors.

[6] Pyo S., Lee J.-I., Kim M.-O., Chung T., Oh Y., Lim S.-C., Park J., Kim J. Batch fabricated flexible tactile sensor based on carbon nanotube-polymer composites. Proceedings of the IEEE 2013 Transducers & Eurosensors XXVII: The 17th International Conference on Solid-State Sensors, Actuators and Microsystems (TRANSDUCERS & EUROSENSORS XXVII).

[7] Drimus A., Kootstra G., Bilberg A., Kragic D. (2014). Design of a flexible tactile sensor for classification of rigid and deformable objects. Robot. Auton. Syst..

[8] Canavese G., Stassi S., Fallauto C., Corbellini S., Cauda V., Camarchia V., Pirola M., Pirri C.F. (2014). Piezoresistive flexible composite for robotic tactile applications. Sens. Actuators A Phys..

[9] Stassi S., Canavese G., Cosiansi F., Gazia R., Fallauto C., Corbellini S., Pirola M., Cocuzza M. (2013). Smart piezoresistive tunnelling composite for flexible robotic sensing skin. Smart Mater. Struct..

[10] Kuchibhatla S.V.N.T., Karakoti A.S., Bera D., Seal S. (2007). One dimensional nanostructured materials. Prog. Mater. Sci..

[11] Lu X., Zhang W., Wang C., Wen T.C., Wei Y. (2011). One-dimensional conducting polymer nanocomposites: Synthesis, properties and applications. Prog. Polym. Sci..

[12] Ding B., Wang M., Wang X., Yu J., Sun G. (2010). Electrospun nanomaterials for ultrasensitive sensors. Mater. Today.

[13] Mokhtari F., Salehi M., Zamani F., Hajiani F., Zeighami F., Latifi M. (2016). Advances in electrospinning: The production and application of nanofibres and nanofibrous structures. Text. Prog..

[14] Wang Y.R., Zheng J.M., Ren G.Y., Zhang P.H., Xu C. (2011). A flexible piezoelectric force sensor based on PVDF fabrics. Smart Mater. Struct..

[15] Shin M., Song J.H., Lim G.H., Lim B., Park J.J., Jeong U. (2014). Highly stretchable polymer transistors consisting entirely of stretchable device components. Adv. Mater..

[16] Wang L., Ding T., Wang P. (2009). Thin flexible pressure sensor array based on carbon black/silicone rubber nanocomposite. IEEE Sens. J..

[17] Sherman R.D., Middleman L.M., Jacobs S.M. (1983). Electron transport processes in conductor-filled polymers. Polym. Eng. Sci..

[18] McLachlan D.S., Blaszkiewicz M., Newnham R.E. (1990). Electrical resistivity of composites. J. Am. Ceram. Soc..

[19] Hu N., Karube Y., Yan C., Masuda Z., Fukunaga H. (2008). Tunneling effect in a polymer/carbon nanotube nanocomposite strain sensor. Acta Mater..

[20] Shih W.-P., Tsao L.-C., Lee C.-W., Cheng M.-Y., Chang C., Yang Y.-J., Fan K.-C. (2010). Flexible temperature sensor array based on a graphite-polydimethylsiloxane composite. Sensors.

[21] Khan S., Tinku S., Lorenzelli L., Dahiya R.S. (2015). Flexible tactile sensors using screen-printed P(VDF-TrFE) and MWCNT/PDMS composites. IEEE Sens. J..

[22] Vatani M., Engeberg E.D., Choi J.-W. (2014). Detection of the position, direction and speed of sliding contact with a multi-layer compliant tactile sensor fabricated using direct-print technology. Smart Mater. Struct..

[23] Dror Y., Salalha W., Khalfin R.L., Cohen Y., Yarin A.L., Zussman E. (2003). Carbon nanotubes embedded in oriented polymer nanofibers by electrospinning. Langmuir.

[24] Celzard A., McRae E., Deleuze C., Dufort M., Furdin G., Marêché J. (1996). Critical concentration in percolating systems containing a high-aspect-ratio filler. Phys. Rev. B.

[25] Balberg I. (1985). Universal percolation-threshold limits in the continuum. Phys. Rev. B.

[26] Huang Z.-M., Zhang Y.-Z., Kotaki M., Ramakrishna S. (2003). A review on polymer nanofibers by electrospinning and their applications in nanocomposites. Compos. Sci. Technol..

[27] Theron A., Zussman E., Yarin A.L. (2001). Electrostatic field-assisted alignment of electrospun nanofibres. Nanotechnology.

[28] Li D., Wang Y., Xia Y. (2003). Electrospinning of polymeric and ceramic nanofibers as uniaxially aligned arrays. Nano Lett..

[29] Katta P., Alessandro M., Ramsier R.D., Chase G.G. (2004). Continuous electrospinning of aligned polymer nanofibers onto a wire drum collector. Nano Lett..

[30] Kim K.W., Lee K.H., Khil M.S., Ho Y.S., Kim H.Y. (2004). The effect of molecular weight and the linear velocity of drum surface on the properties of electrospun poly(ethylene terephthalate) nonwovens. Fibers Polym..

[31] Teo W.E., Ramakrishna S. (2006). A review on electrospinning design and nanofibre assemblies. Nanotechnology.

[32] Sun Z., Deitzel J.M., Knopf J., Chen X., Gillespie J.W. (2012). The effect of solvent dielectric properties on the collection of oriented electrospun fibers. J. Appl. Polym. Sci..

[33] Masin S.C., Zudini V., Antonelli M. (2009). Early alternative derivations of Fechner’s law. J. Hist. Behav. Sci..

